# Short-Term Tinnitus Suppression With Electric-Field Guided rTMS for Individualizing rTMS Treatment: A Technical Feasibility Report

**DOI:** 10.3389/fneur.2020.00086

**Published:** 2020-02-25

**Authors:** Stefan Schoisswohl, Berthold Langguth, Martin Schecklmann

**Affiliations:** Department of Psychiatry and Psychotherapy, University of Regensburg, Regensburg, Germany

**Keywords:** repetitive transcranial magnetic stimulation, tinnitus, neuronavigation, individualized treatment, personalized medicine

## Abstract

**Background:** Past research highlighted the benefits of personalized repetitive transcranial magnetic stimulation (rTMS) for the treatment of chronic subjective tinnitus.

**Objective/Hypothesis:** The objective was to investigate the feasibility of rTMS personalization by identifying individually optimal stimulation parameters in test sessions. Particularly, effectiveness and retest–reliability of different stimulation parameters were examined.

**Methods:** Via electric-field guided rTMS, five patients were stimulated with different frequencies on three positions of the left and right superior temporal gyrus on 2 separate days. After each stimulation, the patients had to evaluate tinnitus loudness and discomfort of the used protocol.

**Results:** Individualization of rTMS was possible in all five patients. Significant lower tinnitus loudness was found for 1 Hz stimulation. Positive correlations between 2 days were observed for hemisphere (left, right), position (mSTG, pSTG), and frequency (1, 10, 20 Hz). High-frequency stimulation produced high discomfort.

**Conclusion:** Personalization of rTMS is considered as feasible. Consistency of parameter-specific tinnitus suppression is demonstrated.

## Introduction

Current research findings in the field of neurostimulation and tinnitus are still ambiguous with respect to the effectiveness of repetitive transcranial magnetic stimulation (rTMS) for the treatment of chronic subjective tinnitus (1). It remains unclear which stimulation parameters, e.g., stimulation positions or frequencies, are most effective. To combat this uncertainty and also to enhance the potential efficacy of rTMS for tinnitus, two promising approaches are provided by the personalization of rTMS treatments. First, usage of neuronavigation or electric-field (e-field) guided rTMS is a putative candidate as coil positions can be defined and tracked in the range of mms over the intended target area by means of individual anatomical MRI scans (2, 3). Second, during test sessions using short stimulations with reduced number of pulses, several different frequencies and positions can be tested in order to detect a patient-specific rTMS protocol for the most appropriate short-term tinnitus suppression. Compared to other neuropsychiatric disorders like depression, tinnitus offers the advantage of an immediate response to an intervention - namely changes in tinnitus loudness. The possibility to briefly suppress tinnitus by single-session rTMS for a short period of time is considered as feasible (4). Identified protocols can then be used for daily treatment. The approach to focus on individual results in tinnitus research (in this case, patient-specific rTMS protocols for short-term tinnitus suppression) was already emphasized by Tyler et al. (5). So far, the concept of rTMS personalization has only been examined in a study by Kreuzer et al. (6). Patients were stimulated throughout test sessions with 1-, 5-, 10-, and 20 Hz, continuous theta burst stimulation along with a sham condition (200 or 50 pulses each) over the left and right dorsolateral prefrontal cortex and left and right temporoparietal junction on both hemispheres. Responders (sham-controlled and stimulation-specific reductions) were identified and stimulated with their most effective prefrontal and temporoparietal rTMS protocol over the course of a 2 week period (4,000 pulses per session). Non-responders received the standard stimulation protocol for the particular brain region. Better improvements in tinnitus-related questionnaires were found for the group that received individualized treatment as compared to the group receiving the standard protocol. This confirms the ability to identify personalized rTMS protocols and suggests the superiority of this approach for the treatment of chronic tinnitus with rTMS.

Following on from the findings of this study, the hereafter reported feasibility trial investigates whether it is possible to personalize e-field guided rTMS and examines the effectiveness and retest–reliability of certain stimulation parameters in short-term tinnitus suppression. For this purpose, three different stimulation positions of the left and right superior temporal gyrus (STG) are stimulated by an e-field guided neuronavigation rTMS system with different frequency protocols on two different days.

## Materials and Methods

### Sample Characteristics

Five patients (1 female) with chronic subjective tinnitus (>6 months tinnitus duration) and rTMS experience were enrolled in this feasibility trial. The mean age was 51.6 years (*SD* = 10.92), all of them were right-handers, two experienced unilateral right-sided tinnitus, whereas three suffered from bilateral tinnitus. Primary inclusion criteria were as follows: age 18–75; subjective chronic tinnitus; no serious medical, neurological, or psychiatric disorders; stable medication; no contraindication for magnetic resonance imaging (MRI); no current tinnitus treatment or participation in other tinnitus-related experiments. All patients gave written informed consent prior to the experiment.

### rTMS Test Session

T1-weighted MRI brain scans conducted by a MAGNETOM 1.5-T scanner (Siemens, Germany) preceded the experiment. Individual anatomical images in combination with an e-field guided neuronavigation rTMS system (NBT System 2; Nexstim Plc., Finland) facilitated a stimulation of the patients' STG with millimeter accuracy. Besides visualization and information on the strength of the induced e-field (V/m) in real time, the usage of an e-field guided rTMS system offered the opportunity to control for the direction of the induced electric current [for a detailed description of e-field guided rTMS, see (7)]. Before the test sessions, the patients' resting motor threshold (RMT) was defined via the stimulation of the left primary motor cortex and simultaneous recordings of motor evoked potentials (MEPs) from the thenar muscles of the right hand. Individual motor hotspots were detected by the administration of single pulses (maximum 30 pulses) over different positions of the left primary motor cortex, until several MEPs with a peak-to-peak amplitude of >50 μV were detectable. The position with the highest amplitude threshold was repeated with the aid of an aiming tool implemented in the neuronavigation system (same coil orientation and tilting). Subsequently, RMT determination was performed by applying single pulses with automatically varying intensity shifts (2.5% steps up and down). The RMT was specified as the minimum stimulation intensity needed to produce MEPs with a minimum of 50 μV amplitude in at least 50% of applied pulses.

Six stimulation positions for rTMS were localized in reference to Sahlsten et al. (3). Stimulation positions consisted of anterior (aSTG), middle (mSTG), and posterior (pSTG) parts of the left and right STG. Anterior parts were localized about 1 cm in posterior direction from the junction of sulcus centralis with the STG. PSTG was set at the temporoparietal junction, whereas the mSTG was selected in the middle of those two points. During the test sessions, patients were stimulated with 200 pulses of 1-, 10-, 20-, and 0.1 Hz (20 pulses, active control condition, no neuroplastic effects expected due to few pulses and long pulse intervals) rTMS with 110% RMT.

Each stimulation was executed with the coil placed in such a way that the direction of the induced e-field was perpendicular to the sulcus of the target area. The order of stimulation frequency, hemisphere, and positions was randomized. After each of the 24 protocols, the patients had to rate the loudness of their tinnitus on a numeric rating scale from 0 to 110% of baseline loudness (0% represents a total absence of tinnitus, 100% no change in tinnitus loudness, whereas 110% signified an increase in the tinnitus loudness by 10%) and the degree of discomfort from 0 to 10 (10 signified intolerability) induced by the rTMS interventions. To investigate reliability, the test session was repeated on a second day with a minimum interval of 1 day and a new randomization (in addition reversed order of hemisphere). The most suitable rTMS protocol for short-term tinnitus suppression was identified individually for each patient with respect to the induced tinnitus loudness reduction as well as discomfort rating of the respective rTMS protocol.

### Statistical Analysis

Tinnitus loudness ratings for all patients were averaged for the stimulation parameters frequency, position, and hemisphere and analyzed with the statistic software SPSS (SPSS ver. 24; IBM, USA). Differences in tinnitus loudness and discomfort ratings for stimulation positions and frequencies were analyzed by Friedmann tests. Significant effects were followed up by Wilcoxon tests. Wilcoxon tests were conducted to examine differences in the stimulation hemisphere. Reliability of stimulation parameters in short-term tinnitus suppression (day 1 and day 2) was analyzed by Kendall's Tau correlations with averaging the parameters of non-interest. Statistical significance was set at *p* ≤ 0.05. Plots were created in R (R version 3.4.3; R Foundation for Statistical Computing, Austria) with the package “ggplot2.”

## Results

**Figure 1:**
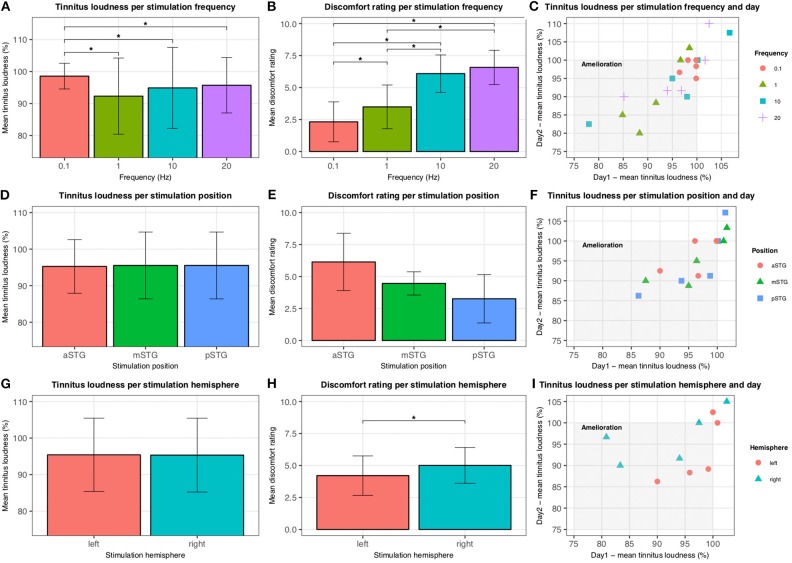
Tinnitus loudness and discomfort ratings. Tinnitus loudness and discomfort ratings for rTMS parameters stimulation frequency **(A,B)**, stimulation position **(D,E)**, and stimulation hemisphere **(G,H)** are illustrated. Error bars represent standard deviations. Significant differences (*p* < 0.05) are highlighted by *. Reliability results for the 2 days are outlined in **(C,F,I)**. Tinnitus amelioration lies within the gray rectangle. Tinnitus loudness ratings for rTMS over the aSTG for day 2 are missing for one patient due to painfulness.

As shown in Table 1, an individual best protocol for brief tinnitus suppression could be identified for each patient. Statistical analysis showed a significant effect of frequency [χ^2^(3) = 9.98, *p* = 0.02]. *Post hoc* tests revealed significant lower tinnitus loudness ratings of 1 Hz compared to 0.1 Hz (z = −2.02, *p* = 0.04), 10 Hz (z = −2.02, *p* = 0.04), as well as 20 Hz (z = −2.02, *p* = 0.04) with no differences between the other contrasts (all *p*-values > 0.05). No significant effects were found for stimulation position [χ^2^(5) = 1.60, *p* = 0.45] and hemisphere (z = −0.67, *p* = 0.50) (compare Figures 1A,D,G). Statistical tests for the discomfort ratings exposed a significant effect of frequency [χ^2^(5) = 9.98, *p* = 0.02]. *Post hoc* Wilcoxon tests revealed significant differences between 0.1 and 1 Hz (z = −2.04, *p* = 0.04), 0.1 and 10 Hz, 0.1 and 20 Hz, 1 and 10 Hz, as well as 1 and 20 Hz (z = −2.02, *p* = 0.04). The sham condition followed by 1 Hz produced the least discomfort ratings. Further analysis indicated significant lower discomfort levels for left hemispheric rTMS (z = −2.02, *p* = 0.04). Near-significant differences were found for the stimulation positions [χ^2^(5) = 5.20, *p* = 0.07] (compare Figures 1B,E,H). Tests for loudness rating reliability discovered a positive correlation between day 1 and day 2 for the left and right hemispheres (*r*_τ_ = 0.80, *p* = 0.05) and also for the stimulation positions mSTG (*r*_τ_ = 0.80, *p* = 0.05) and pSTG (*r*_τ_ = 1.00, *p* = 0.05). Likewise, significant positive correlations were found for the frequencies 1 Hz (*r*_τ_ = 0.80, *p* = 0.05), 10 Hz (*r*_τ_ = 0.80, *p* = 0.05), and 20 Hz (*r*_τ_ = 0.95, *p* = 0.05). Figures 1C,F,I illustrate individual tinnitus loudness ratings for stimulation frequency, position, and hemisphere over the 2 days.

**Table 1:** Individual rTMS protocol per patient.

	**Patient no**.				
	**1**	**2**	**3**	**4**	**5**
Stimulation intensity (%)	36	24	51	30	30
Hemisphere	Right	Left	Left	Right	Right
Position	mSTG	pSTG	pSTG	aSTG	pSTG
Frequency (Hz)	1	1	10	1	1
Tinnitus loudness (% from baseline loudness)	80	90	50	80	50
Discomfort rating of rTMS protocol	3	1	3	2	0

## Conclusion

The aim of this study was to examine if a personalization of rTMS with e-field guided neuronavigation is feasible and reliable. We tested the impact of various stimulation parameters (position, frequency, and hemisphere) on short-term tinnitus suppression. Since none of the patients reported superior tinnitus reduction for the sham condition, together with the possibility to identify an individual protocol for each patient, customization of rTMS protocols by performing test sessions with different protocols is considered as feasible.

With respect to retest–reliability for stimulation parameters, we demonstrated consistent tinnitus loudness ratings for hemisphere (left, right), stimulation position (mSTG, pSTG), and frequency (1, 10, 20 Hz) in tinnitus suppression. Therefore, the consistency of parameter-specific tinnitus suppression can be assumed.

We also observed a significant group effect of the protocol with superior effects for 1 Hz suggesting the superiority of 1 Hz over the other tested frequencies (10, 20 Hz) as a general effect, which does not depend on individual differences in susceptibility to rTMS effects.

Discomfort ratings indicate that stimulations over the aSTG (one patient dropout; aSTG < mSTG < pSTG) of the right hemisphere and with high-frequency protocols (0.1 < 1 < 10 < 20 Hz) as most unpleasant. These discoveries underline the usefulness of test sessions in order to identify an rTMS protocol with the best tolerability and the best tinnitus suppression. On the other hand, the influence of local side effects on loudness reduction cannot be excluded. The cooling noise of the TMS coil made it rather difficult for some patients to accurately rate loudness changes of their tinnitus during the test sessions. It is conceivable that tinnitus loudness ratings and thereby parameter effectiveness would be different with a silent coil. Since the same coil was used for the whole trial, reliability results should not be affected. However, future test session trials should strive for bigger sample sizes and implementation of silent or uncooled TMS coils, and focus either on other brain regions or one stimulation position to tackle the huge testable parameter space in rTMS.

## Data Availability Statement

The datasets generated for this study are available on request to the corresponding author.

## Ethics Statement

The studies involving human participants were reviewed and approved by ethic commission of the University of Regensburg. The patients/participants provided their written informed consent to participate in this study. This trial was approved by the local ethics committee of the University of Regensburg, Germany (17-820-101).

## Author Contributions

MS, BL, and SS designed the study. MS and SS carried out the experiment and analyzed the data. SS wrote the main manuscript. All authors contributed to and reviewed the manuscript.

### Conflict of Interest

The authors declare that the research was conducted in the absence of any commercial or financial relationships that could be construed as a potential conflict of interest.
